# Editorial: From nutrients, food matrices, bioactive compounds, to microbiota: exploring the path to optimal human health - Congress farm to fork: our food, our health, our future

**DOI:** 10.3389/fnut.2025.1738451

**Published:** 2025-11-21

**Authors:** Inês Brandão, Wisnu Adi Wicaksono, Ana Faria, Patrícia Coelho, Christophe Espírito Santo

**Affiliations:** 1 Centro de Apoio Tecnológico Agro Alimentar (CATAA), Castelo Branco, Portugal; 2 Centre for Functional Ecology, Associate Laboratory TERRA, Department of Life Sciences, University of Coimbra, Coimbra, Portugal; 3 Institute of Environmental Biotechnology, Graz University of Technology, Graz, Austria; 4 Faculdade de Ciências Médicas, Nutrition & Metabolism, NOVA Medical School, NMS, FCM, Universidade NOVA de Lisboa, Lisboa, Portugal; 5 Faculdade de Ciências Médicas, CHRC, NOVA Medical School, NMS, FCM, Universidade NOVA de Lisboa, Lisboa, Portugal; 6 Sport Physical Activity and Health Research & Innovation Center (SPRINT) - Polytechnic University of Castelo Branco, Castelo Branco, Portugal

**Keywords:** food matrices, bioactive compounds, fibre, microbiota, sustainable foods

Inspired by the *Farm to Fork: Our Food, Our Health, Our Future* Congress (Castelo Branco, Portugal), this Research Topic examines the final farm-to-fork stage, where nutritional quality, bioactive compounds, edible microbiota and food matrices intersect to shape human health; the papers were not presented at the Congress but reflect its themes.

The studies gathered in this Research Topic tell a coherent story: diet acts both through content and structure. Not simply over grams of a nutrient, but through the way food matrices and microbial communities meet and modulate host physiology. Read together, these papers advance four main practical ideas. First, microstructure guides which microbes and metabolites prevail. Second, immune and metabolic function can shift even when community composition seems unchanged, so outcomes that capture function deserve equal footing with taxonomic profiles. Third, intact matrices shape risk and benefit, calling for matrix-aware guidance aligned with Farm-to-Fork.

Two contributions on fibers and resistant starch (RS) make the “structure matters” point concrete. Tang et al. test an RS3 from *Canna edulis* (CE) and show that simulated digestion increases crystallinity and surface roughness, explaining enzyme resistance and readiness for colonic fermentation. In fecal cultures, the post-digestion material lowered pH, increased short-chain fatty acids, expanded *Bifidobacterium* and suppressed *Escherichia–Shigella*, whereas fructo-oligosaccharide acidified more deeply and reduced diversity. CE thus offers a credible functional-food candidate and a clear example of microstructure steering fermentation.

Chong et al. take the next step with upcycled fruit fibers and shows clear, source-specific responses. In a short, randomized intervention, green banana (GBP) and pineapple (PFP) powders (both enriched in insoluble fiber) were well-tolerated, improved bowel regularity, increased beneficial taxa and boosted microbial functions (including SCFA and vitamin-linked pathways). Effects were source-specific and subgroup-dependent, yet both interventions suppressed *Escherichia–Shigella* and raised *Bifidobacterium* without loss of diversity. These results make a practical case for circular, food-grade fibers that shift fermentation toward health-relevant metabolites, though longer, diet-controlled trials across different population groups are needed to test durability and clinical impact.

Fermentation emerges here as a design tool rather than a culinary afterthought. Zhu et al. show that a practical *Levilactobacillus brevis* process can deliver a GABA-enriched yogurt under standard dairy conditions—no new equipment, sensory quality intact, live cultures maintained, and GABA stable during chilled storage. Mixed co-fermentation with *Streptococcus thermophilus* further raises GABA while keeping acidity in the commercial range. In effect, fermented dairy is positioned as a carrier for neuroactive metabolites relevant to blood pressure, stress and sleep—areas where modest, food-based effects matter. The next step is clear: dose–response trials that track clinical endpoints and confirm that bioactivity persists across shelf life. More broadly, it shows how strain, process and matrix can be tuned together to deliver function, not just a label claim.

In the same vein, and continuing to harness microorganisms as cell factories for potentially functional bioactive food ingredients, Yu and colleagues screened *Lacticaseibacillus paracasei* ZFM54 for strong exopolysaccharide (EPS) production and optimized its fermentation. EPS effectively reduced *Helicobacter pylori* colonization to the epithelial AGS cells, recovering cell morphology, and alleviated gastritis in infected mice, partly restoring gastric microbiota structure, and shifted cytokine expression toward a less inflammatory profile. Noteworthy, as antibiotic resistance rises, a structure-defined biopolymer with antimicrobial and immunomodulatory actions is notable and these results provide a basis to explore food applications (e.g., fermented dairy), pending human safety and pharmacokinetic data.

Safety is treated with the same emphasis on function. Fischer et al. exposed a simplified gut community co-cultured with immune cells to common chemical mixtures (bisphenols, PFAS). Immune-modulating capacity changed despite little movement in alpha- or beta-diversity, challenging the use of composition alone as a proxy for health. Preclinical screens and human studies should therefore register functional readouts (e.g., immune phenotypes, SCFA and bile-acid panels, barrier measures) alongside taxonomy, and validate *in vitro* against *in vivo* outcomes.

The matrix argument is illustrated in the narrative review by Eugénio et al. which revisit cheese, a food with a long cultural history and a complex food matrix. Synthesizing recent observational and interventional evidence, they conclude that cheese, particularly in its intact matrix, does not appear linked to adverse cardiometabolic outcomes and may confer protection. Plausible mechanisms include calcium-mediated lipid handling, microbial peptides with ACE-inhibitory activity, SCFA production, polar lipids and an active microbiota. Methodologically, trials must control fat and salt while varying microstructure; matrix-matched, longer randomized trials are needed to show causality and inform new matrix-based dietary guidelines.

What, then, should the field do differently? First, put function on par with composition. Register immune and metabolite endpoints up front in protocols, not as exploratory afterthoughts. Second, describe the materials properly (e.g., for fibers that means reporting crystallinity, branching, particle size, etc.). Without these, reproducibility and synthesis suffer. Third, lengthen and power trials appropriately. Many diet studies are too short to capture adaptation; according to the literature, 8–12 weeks seems to a sensible minimum for fiber and resistant starch, with dose–response built in, background diet recorded or controlled, and analyses stratified by age, BMI and baseline microbiome. Fourth, test matrices head-to-head. For example, in cheese studies hold fat and salt constant and vary microstructure to isolate matrix effects. Finally, align health impact with sustainability by clinically evaluating upcycled ingredients and communicating structure and function clearly to consumers.

It is also worth noting the limits of what is currently known. Short interventions and modest doses may underestimate effect sizes or fail to show durability. *In vitro* models that highlight functional shifts need *in vivo* confirmation, although they provide an ethical and standardisable first filter. Heterogeneity in medication use, lifestyle and baseline microbiota will continue to modulate response; that is an argument for a priori stratification. And composition-only readouts can create misplaced confidence; functional outcomes help prevent that.

Why does this agenda matter for Farm-to-Fork? It connects foods people actually eat, ingredients that use resources better, and outcomes that change population-level risk. Banana and pineapple fibers illustrate clinically relevant upcycling; GABA yogurt shows an everyday matrix carrying a specific bioactive; EPS work suggests a non-antibiotic adjunct in gastric infection; and the cheese review reminds us matrices can gate risk as much as nutrients. None of these advances rest on a single super-ingredient; they rest on structure and context.

In sum, the papers in this Topic support a practical shift in nutrition science: from counting to understanding, from lists of components to the ways in which matrices and microbes interact with the host. By pre-registering functional outcomes, giving structural features the same attention as nutrient amounts, and adopting longer, stratified trials, researchers can judge foods on performance rather than composition alone. The result benefits health and sustainability and fits the Farm-to-Fork agenda.

